# 

**Published:** 1894-05

**Authors:** 


### CONTENTS.

ORIGINAL ARTICLES—

Teething. By H. McHatton, M.D.. Macon, Ga................................ 233
Endometritis—Its Pathology, Symptomatology and Treatment. By William W. Stewart, M.D., Columbus, Ga....................................... 244
A Case of Catarrhal Pneumonia in Childhood. By Albert S. Miller, N.G., M D., Athens, Texas ........................................... 252
Nephrectomy for Sarcoma, with Report of Case and Exhibition of Specimen. By J. B. S. Holmes, M.D., Atlanta, Ga........................ 256

SOCIETY REPORTS—

Georgia State Medical Association............................................. 259

BOOK REVIEWS—

A Practical Treatise on Medical Diagnosis, for Students and Physicians. By John H. Musser, M.D., Philadelphia ............................ 273
Essentials of Physics. By Fred. J. Brockway, M.D., New York......... 274
The Year Book of Treatment, 1894............................................ 274

SELECTIONS AND ABSTRACTS—

Sulfonal in the Treatment of the Insane........................... ...... ... ...... 275

A Convenient Antiseptic Uunder-Sheet in Child-Birth... ................. 275

EDITORIAL—

Mr. Gladstone on the Medical Profession......................... ........ ..... 276

EDITORIAL NOTES................................................................ ............ 278

PRESCRIPTION DEPARTMENT............................... ................282, 283

Inanition in Children—Tooth and Mouth Wash—Acute Coryza—For Shock Following Abdominal Operations – Chronic Gastric Ulcer—Hydrobromic Cough-mixture—For Eczema, Herpes, Other Skin Affections—For Obesity and Fatty Degeneration of the Heart—Chronic Bronchitis—Alcoholism.

SPECIAL NOTES..... ......... ........ ..................................284, 286, 288, 290

